# Stochastic modelling of infectious diseases for heterogeneous populations

**DOI:** 10.1186/s40249-016-0199-5

**Published:** 2016-12-22

**Authors:** Rui-Xing Ming, Ji-Ming Liu, William K. W. Cheung, Xiang Wan

**Affiliations:** 1 School of Statistics & Mathematics, Zhejiang Gongshang University, Hangzhou, China; 2 Department of Computer Science, Hong Kong Baptist University, Kowloon Tong, Hong Kong; 3 Department of Computer Science & Institute of Theoretical and Computational Study, Hong Kong Baptist University, Kowloon Tong, Hong Kong

**Keywords:** Epidemiology, Stochastic model, Surveillance system, Spread pattern

## Abstract

**Background:**

Infectious diseases such as SARS and H1N1 can significantly impact people’s lives and cause severe social and economic damages. Recent outbreaks have stressed the urgency of effective research on the dynamics of infectious disease spread. However, it is difficult to predict when and where outbreaks may emerge and how infectious diseases spread because many factors affect their transmission, and some of them may be unknown.

**Methods:**

One feasible means to promptly detect an outbreak and track the progress of disease spread is to implement surveillance systems in regional or national health and medical centres. The accumulated surveillance data, including temporal, spatial, clinical, and demographic information can provide valuable information that can be exploited to better understand and model the dynamics of infectious disease spread. The aim of this work is to develop and empirically evaluate a stochastic model that allows the investigation of transmission patterns of infectious diseases in heterogeneous populations.

**Results:**

We test the proposed model on simulation data and apply it to the surveillance data from the 2009 H1N1 pandemic in Hong Kong. In the simulation experiment, our model achieves high accuracy in parameter estimation (less than 10.0 *%* mean absolute percentage error). In terms of the forward prediction of case incidence, the mean absolute percentage errors are 17.3 *%* for the simulation experiment and 20.0 *%* for the experiment on the real surveillance data.

**Conclusion:**

We propose a stochastic model to study the dynamics of infectious disease spread in heterogeneous populations from temporal-spatial surveillance data. The proposed model is evaluated using both simulated data and the real data from the 2009 H1N1 epidemic in Hong Kong and achieves acceptable prediction accuracy. We believe that our model can provide valuable insights for public health authorities to predict the effect of disease spread and analyse its underlying factors and to guide new control efforts.

**Electronic supplementary material:**

The online version of this article (doi:10.1186/s40249-016-0199-5) contains supplementary material, which is available to authorized users.

## Multilingual abstracts

Please see Additional file 1 for translations of the abstract into the five official working languages of the United Nations.

## Background

Infectious diseases remain a major cause of morbidity and mortality worldwide, triggering immeasurable loss in many societies. Most people may still have a fresh memory of the H1N1 outbreak in 2009, which brought pictures of empty streets and people wearing face masks and collectively caused at least 12799 deaths according to the World Health Organization (WHO) report [1]. The H1N1 pandemic calls for research on accurately modelling the spread dynamics of an infectious disease, which offers a practically useful means for policy makers to evaluate the potential effects of intervention strategies [2–4].

Mathematical models of the spread of infectious diseases are an important tool for investigating and quantifying the spread dynamics because direct experimental study on the spread of disease among humans is not ethical. Although the subjects involved in different epidemics may be different, many can be modeled by the popular Susceptible-Infected-Recovered (SIR) models [5–7], which study the spread of infectious diseases by tracking the number (S) of people susceptible to the disease, the number (I) of people infected with the disease, and the number (R) of people who have recovered from the disease. Three assumptions are made: (1) the total population *N*=*S*(*t*)+*I*(*t*)+*R*(*t*) is fixed at any time *t*; (2) those who have recovered from the disease are forever immune; and (3) those who have not had the disease are equally susceptible, and the probability of their contracting the disease at time *t* is proportional to the product of *S*(*t*) and *I*(*t*). Based on these assumptions, the SIR model defines a set of three ordinary differential equations for S(t), I(t), and R(t):

1 $$\begin{array}{@{}rcl@{}} {dS}/{dt} & = & - \beta S(t) I(t)  \\ {dI}/{dt} & = & \beta S(t)I(t) - k I(t)  \\ {dR}/{dt} & = & k I(t). \end{array} $$

Here, *β*≥0 is the effective transmission rate and *k*≥0 is the recovery rate. Because the SIR-based models are well presented in the literature, herein, we omit a verbose introduction of these models. Readers with an interest in such a topic can find the details in [5–7].

The SIR-based models and its variants have proven to be quite useful in the study of the spread dynamics of infectious diseases [8–10]. In [11–13], the progression of disease spread is characterized by tracking the number of *S*_*t*_ with a chain binomial model. The number of susceptible members *S*_*t*+△*t*_ (△*t* represents the infectious period of the disease and is always chosen to be 1/*k*) at time *t*+△*t* is a binomial random variable that depends on *S*_*t*_ and *I*_*t*_ *α*, *S*_*t*+△*t*_∼*B* *i* *n*(*S*_*t*_,1−*I*_*t*_ *α*), which provides a recursive relationship between *S*_*t*+△*t*_ and *S*_*t*_ and produces a formal stochastic process. However, the power of these models is mainly limited to uniform and homogeneous populations or populations with infinite size and homogeneous interactions. In many cases, the actual spread of infectious diseases occurs in a diverse or dispersed population. To study the spread of infectious diseases in heterogeneous populations, people usually divide a population into subpopulations that differ from each other. Sub-populations can be determined on the basis of social, cultural, economic, demographic, and geographic factors. Next, besides the dynamics of the internal spread within a subpopulation, the transmission dynamics between subpopulations should also be considered in the study of epidemic spreading.

Network-based epidemic modelling represents a popular approach for heterogeneous populations in which the nodes in the network correspond to sub-populations, and the links indicate the neighboring relationships. Many network-based models have been proposed, including patch models [14–16], distance-transmission models [17], and multi-group models [18, 19]. However, these models require knowledge of every individual (or host) and all relationships between individuals, which may be not achievable due to information privacy-related restrictions and the high cost of subject recruitment. To overcome the difficulties of collecting data, researchers have investigated several types of computer-generated networks in the context of disease spread in population-scale studies [20–24]. Grassberger first studied the dynamics of infectious diseases that propagate on regular networks using the percolation theory [25]. Recent studies have revealed that many real-world networks, including social networks in which infectious diseases propagate, are either small-world [26] or scale-free [27] rather than regular or random, as thought previously [28]. Because the underlying structures of networks will influence the effect that the dynamics of epidemics will have on them, researchers, such as Pastor-Satorras and Vespignani, have made many contributions to critical value analysis of typical epidemics on different types of complex network [23, 24, 29]. On the basis of the mean-field theory, they found that compared with homogeneous networks, scale-free networks are fragile to the invasion of infectious diseases, computer viruses, or any other type of negative epidemics.

Epidemics have also been studied in various disciplines. Sociologists are concerned with the diffusion of rumors or innovation on social networks [30]; economists have studied viral marketing and recommendation strategies by considering both cascading dynamics and the network effects of vital nodes [31]; and computer scientists are interested in how some topics can quickly cascade in virtual blog spaces and how their propagation trends [32, 33].

Although network-based studies have contributed to the modelling of disease and/or information dynamics, some models make a strong assumption that the structures of underlying networks over which epidemics spread are known beforehand. In the real world, however, the structures of underlying diffusion networks are not known directly. Many others assume the availability of information about the interactions occurring between individuals [34–37] that are often not valid in the context of disease spread. What may be obtained is only the time at which particular sub-populations become infected, but not how they become infected, nor how they affect their neighboring areas. Moreover, the underlying structures of networks will greatly influence the dynamics of infectious disease spread.

Since the emergence of the H1N1 influenza pandemic in April 2009, its underlying dynamics have been of great public health interest, and many approaches for its study have been proposed [14, 38–41]. Most of them are based on the classic SIR model. For example, Birrell et al. [40] provided an age structure-based compartmental model with a Bayesian synthesis of multiple evidence sources to reveal substantial changes in contact patterns throughout the epidemic. Besides of the compartmental models, other mathematical models are also used to describe the transmission dynamics [3, 42–47]. The chain binomial model was used to calculate the household secondary attack rates to measure the transmissibility of the 2009 H1N1 influenza pandemic by Lessler et al. [44] and Klick et al. [45]. Yang et al. [46] constructed a model based on chains of infections and used the infection hazard function and survival function to study the 2009 H1N1 influenza pandemic. Ferguson et al. [3] and Cauchemez et al. [42, 43] incorporated other factors, such as household risk, within-school risk, and community risk, in the study of infection spread and found out that younger age groups under 19 years old were more susceptible than older age groups. Jin et al. [47] formulated an epidemic model of influenza A based on networks and calculated the basic reproduction number and studied the effects of various immunization schemes. However, this work required that the individual contact pattern be provided. Nonetheless, none of the aforementioned approaches takes spatial heterogeneity into consideration in the study of disease spread.

Recently, an outbreak of Ebola virus disease (EVD) swept across parts of West Africa from March 2014 to April 2015. By June 10, 2015, WHO had reported 27,237 confirmed, probable, or suspected cases in three countries with 11,158 deaths [48]. This epidemic received extensive research attention on its dynamics of spread [49–57] (for further references in the review article [58]). To name a few, Chowell et al. found that district-level Ebola virus disease outbreaks in West Africa follow polynomial-based growth in time instead of the exponential growth that describes the progress of many infectious disease epidemics [52]. Fisman et al. used a simple, two parameter mathematical model to characterize epidemic growth patterns in the 2014 Ebola outbreak [53]. Webb et al. proposed a variant of the classic SIR model with three extra groups, incubating, contaminated and isolated, which can provide a more accurate prediction for the future incidences [56]. Carroll et al. used a deep sequencing approach to gain insight into the evolution of the Ebola virus (EBOV) in Guinea from the ongoing West African outbreak. The viral sequence data can be combined with epidemiological information to retrospectively test the effectiveness of control measures, and provides an unprecedented window into the evolution of an ongoing outbreak of viral haemorrhagic fever [57].

To accurately predict when and where outbreaks will occur, a feasible means is to deploy manual or electronic surveillance systems through regional or national public health and medical organizations [59]. Most of the surveillance data accumulated from such systems contains temporal, spatial, clinical, and demographic information. For instance, Telehealth Ontario is a teletriage helpline that is available free to all Ontario residents, which allows those with suspected infections to connect with experts who can assess their symptoms. The records of such calls provide valuable information on which individual from where was possibly infected and by which type of disease at what time. In this paper, we address the problem of modelling disease spread dynamics in heterogeneous populations from temporal-spatial surveillance data. We analyse the role of heterogeneity in a stochastic epidemic model on a two-dimensional lattice. Within a particular sub-population, the speed of spread is controlled by a single parameter, the transmissibility of the pathogen between individuals. Between sub-populations, the transmissibility becomes a random variable drawn from a probability distribution. Our work differs from existing studies in some fundamental ways, in light of the unique nature of infectious disease diffusion dynamics. Our results have practical implications for the analysis of disease control strategies in realistic heterogeneous epidemic systems.

## Methods

In this work, we propose a stochastic model to study the dynamics of infectious disease spread in heterogeneous populations from temporal-spatial surveillance data. We divide the whole population into *m* sub-populations on the basis of geographic regions. In the following, we use *S*_*i*_(*t*),*I*_*i*_(*t*), and *R*_*i*_(*t*) to denote the number of susceptible, infected, and recovered people, respectively, at time *t* in region *i*, *i*=1,2,⋯,*m* and *t*∈[0,*T*].

### Stochastic model

Classic SIR-based modelling of infectious diseases assumes that the population is well-mixed. To take the role of heterogeneity into consideration, we use an alternative approach to model the dynamics of infectious disease spread. First, the classic SIR model (Eq. (1)) studies the change in the numbers of peoples in the three groups. In reality, the change in the number of the infected people is the major concern of society. Second, in many epidemics or pandemics such as H1N1 and SARS, the number of infected people *I*_*i*_(*t*) is relatively small compared to the whole subpopulation *S*_*i*_(*t*). Therefore, we may consider *S*_*i*_(*t*) as a constant to simplify the modelling of the change in the number of infected people *I*(*t*), for which we propose the following stochastic differential equation:

2 $$\begin{array}{@{}rcl@{}} \mathrm{d}I_{i}(t)=(\alpha+\delta_{i}I_{i}(t))\mathrm{d}t+\sigma_{i}\mathrm{d}B_{i}(t), \end{array} $$

where *α* is a parameter that measures the auto-recovery rate of one particular infectious disease, which is usually considered as a constant among sub-populations, *δ*_*i*_ is the parameter that measures the different disease transmissibility in different subpopulations, *σ*_*i*_>0 is the diffusion parameter that measures the disease spread from neighbors, and *B*_*i*_(*t*) is a standard Brownian motion. It is worth noting that we assume the parameter *δ*_*i*_≠0 for technical purposes, and the results in the case of *δ*_*i*_=0 can be achieved with *δ*_*i*_→0.

Comparing our model in Eq. (2) with the classic model in Eq. (1), we can see that they both capture the situation in which the change in the number of infected people has a positive relationship with the total number of infected people, which means that the more infected people there are, the more people will get infected. There are two key differences between these two models: first, the key factor (*β* *S*_*i*_(*t*)−*k*) associated with the disease spread in Eq. (1) is replaced with a single parameter *δ*_*i*_ in Eq. (2), which can be used to analyse the role of heterogeneity in the disease spread; and second, Eq. (2) takes the neighboring relationships into consideration to study the dynamics of the disease spread among different sub-populations.

By Ito formula, the solution of Eq. (2) is given by

3 $$\begin{array}{@{}rcl@{}} &I_{i}(t)=I_{i}(0)e^{\delta_{i}t}+\frac{\alpha}{\delta_{i}}(e^{\delta_{i}t}-1)\\ &\quad+\sigma_{i} e^{\delta_{i}t}{\int_{0}^{t}}e^{-\delta_{i}s}\mathrm{d}B_{i}(s). \end{array} $$

Notice that for any fixed *t*, ${\int _{0}^{t}}e^{-\delta _{i}s}\mathrm {d}B_{i}(s)$ is a normal random variable with

4 $$\begin{array}{@{}rcl@{}} E\left[\int_{0}^{t}e^{-\delta_{i}s}\mathrm{d}B_{i}(s)\right]=0, \\ Var\left[\int_{0}^{t}e^{-\delta_{i}s}\mathrm{d}B_{i}(s)\right]=\frac{1-e^{-2\delta_{i}t}}{2\delta_{i}}. \end{array} $$

Thus, for any fixed *t*, *I*_*i*_(*t*) is a normal random variable with

5 $$\begin{array}{@{}rcl@{}} E[I_{i}(t)]=I_{i}(0)e^{\delta_{i}t}+\frac{\alpha}{\delta_{i}}\left(e^{\delta_{i}t}-1\right) \end{array} $$

and

6 $$\begin{array}{@{}rcl@{}} Var[I_{i}(t)]=\frac{{\sigma_{i}^{2}}}{2\delta_{i}}\left(e^{2\delta_{i}t}-1\right). \end{array} $$

There are three cases of being interested for parameter *α*:

- *α*>−*I*_*i*_(0)*δ*_*i*_
  In this case, *E*[*I*_*i*_(*t*)] tends to infinity as *t* goes to infinity, which implies that all people in that region will be infected if the time is long enough.
- *α*=−*I*_*i*_(0)*δ*_*i*_
  In this case, the pandemic or epidemic will reach a state of equilibrium.
- *α*<−*I*_*i*_(0)*δ*_*i*_
  In this case, *E*[*I*_*i*_(*t*)] will reach 0 at some time $t = \hat {t}$ and go to negative infinity as *t* goes to infinity, which implies the pandemic or epidemic will end at time $\hat {t}$.

### Parameter estimation

To estimate the parameters in our proposed stochastic model from the surveillance data, we need to divide the interval [0,*T*] into *n* subintervals, [*t*_0_,*t*_1_], [*t*_1_,*t*_2_], ⋯, [*t*_*n*−1_,*t*_*n*_], where 0=*t*_0_<*t*_1_<*t*_2_<⋯<*t*_*n*_=*T*. Denote △*t*(*k*)=*t*_*k*+1_−*t*_*k*_, △*B*_*i*_(*k*)=*B*_*i*_(*t*_*k*+1_)−*B*_*i*_(*t*_*k*_), △*I*_*i*_(*k*)=*I*_*i*_(*t*_*k*+1_)−*I*_*i*_(*t*_*k*_), *k*=0,1,⋯,*n*−1. Then Eq. (2) is rewritten as

7 $$\begin{array}{@{}rcl@{}} \triangle I_{i}(k)=(\alpha+\delta_{i}I_{i}(t_{k}))\triangle t(k)+\sigma_{i}\triangle B_{i}(k). \end{array} $$

It is easy to see that $\triangle I_{i}(k)|I_{i}(t_{k})\sim N\big ((\alpha +\delta _{i}I_{i}(t_{k}))\triangle t(k),{\sigma _{i}^{2}}\triangle t(k)\big)$. Let *θ*_*i*_=(*α*,*δ*_*i*_,*σ*_*i*_). Then the transition density of the process {*I*_*i*_(*t*);*t*≥0} is

8 $$\begin{array}{@{}rcl@{}} &&p_{\theta_{i}}(s+t,y|s,x)\\ &=&\frac{1}{\sqrt{2\pi t{\sigma_{i}^{2}}}}\exp\left\{-\frac{(y-x-(\alpha+\delta_{i}x)t)^{2}}{2t{\sigma_{i}^{2}}}\right\}. \end{array} $$

Hence, the likelihood function is given by

9 $$\begin{array}{@{}rcl@{}} &&f(\theta_{i}|I_{i})\triangleq f\left(\theta_{i}|I_{i}(t_{k}),0\leq k\leq n-1\right)\\&=&I_{i}(0)\left(\frac{1}{2{\pi\sigma_{i}^{2}}}\right)^{\frac{n}{2}}\prod\limits_{k=0}^{n-1}\frac{1}{\triangle t(k)} \\ &&\exp\left\{-\frac{(\triangle I_{i}(k)-(\alpha+\delta_{i}I_{i}(t_{k}))\triangle t(k))^{2}}{2\triangle t(k){\sigma_{i}^{2}}}\right\}.\\ \end{array} $$

Consequently, the log-likelihood function is

10 $$\begin{array}{@{}rcl@{}} &&\log f(\theta_{i}|I_{i})\varpropto-\frac{n}{2}{\log\sigma_{i}^{2}}\\ &&-\sum\limits_{k=0}^{n-1}\frac{\left(\triangle I_{i}(k)-(\alpha+\delta_{i}I_{i}(t_{k}))\triangle t(k)\right)^{2}}{2\triangle t(k){\sigma_{i}^{2}}}. \end{array} $$

Let

11 $$\begin{array}{@{}rcl@{}} u_{i1}&=&\frac{1}{T}\sum\limits_{k=0}^{n-1}I_{i}(t_{k}), \end{array} $$

12 $$\begin{array}{@{}rcl@{}} u_{i2}&=&\frac{1}{T}\sum\limits_{k=0}^{n-1}I_{i}(t_{k+1}), \end{array} $$

13 $$\begin{array}{@{}rcl@{}} u_{i11}&=&\frac{1}{T}\sum\limits_{k=0}^{n-1}{I_{i}^{2}}(t_{k}), \end{array} $$

14 $$\begin{array}{@{}rcl@{}} u_{i12}&=&\frac{1}{T}\sum\limits_{k=0}^{n-1}I_{i}(t_{k})I_{i}(t_{k+1}), \end{array} $$

15 $$\begin{array}{@{}rcl@{}} u_{i1\Delta}&=&\frac{1}{T}\sum\limits_{k=0}^{n-1}I_{i}(t_{k})\triangle t(k), \end{array} $$

16 $$\begin{array}{@{}rcl@{}} u_{i11\Delta}&=&\frac{1}{T}\sum\limits_{k=0}^{n-1}{I_{i}^{2}}(t_{k})\triangle t(k), \end{array} $$

17 $$\begin{array}{@{}rcl@{}} u_{i11\Delta^{-1}}&=&\frac{1}{T}\sum\limits_{k=0}^{n-1}{I_{i}^{2}}(t_{k})(\triangle t(k))^{-1}, \end{array} $$

18 $$\begin{array}{@{}rcl@{}} u_{i12\Delta^{-1}}&=&\frac{1}{T}\sum\limits_{k=0}^{n-1}I_{i}(t_{k})I_{i}(t_{k+1})(\triangle t(k))^{-1}, \end{array} $$

19 $$\begin{array}{@{}rcl@{}} u_{i22\Delta^{-1}}&=&\frac{1}{T}\sum\limits_{k=0}^{n-1}{I_{i}^{2}}(t_{k+1})(\triangle t(k))^{-1}. \end{array} $$

We have the estimator of *θ*_*i*_ as follows:

20 $$\begin{array}{@{}rcl@{}} \widehat{\delta}_{i}&=&\frac{u_{i12}-u_{i11}-u_{i2}u_{i1\Delta}+u_{i1}u_{i1\Delta}}{u_{i11\Delta}-u_{i1\Delta}^{2}}, \end{array} $$

21 $$\begin{array}{@{}rcl@{}} \widehat{\alpha}&=&u_{i2}-u_{i1}-\widehat{\delta}_{i}u_{i1\Delta}, \end{array} $$

22 $$\begin{array}{@{}rcl@{}} \widehat{{\sigma^{2}_{i}}}&=&Tn^{-1}\left\{u_{i22\Delta^{-1}}-2u_{i12\Delta^{-1}}+u_{i11\Delta^{-1}}\right.\\ && -(u_{i2}-u_{i1})^{2}+\left[({u_{i2}}-{u_{i1}})u_{i1\Delta}\right.\\ && \left.\left.-(u_{i12}-u_{i11})\right]\widehat{\delta}_{i}\right\}. \end{array} $$

It is obviously to see that $\widehat {\alpha }$ is not a bona fide estimator of *α*, because only the information of {*I*_*i*_(*t*);0≤*t*≤*T*} is used to estimate *α*. A good estimator should pool all the information {*I*_*i*_(*t*);0≤*t*≤*T*} (*i*=1,2,⋯,*m*). There are two ways to find the pool estimator. The first way is to approximate *α* by pooling all $\widehat {\alpha }_{i}$ as follows:

23 $$\begin{array}{@{}rcl@{}} \widehat{\alpha}&=&m^{-1}\sum\limits_{i=1}^{m}\widehat{\alpha_{i}}, \end{array} $$

24 $$\begin{array}{@{}rcl@{}} \widehat{\alpha_{i}}&=& u_{i2}-u_{i1}-\widehat{\delta}_{i}u_{i1\Delta}. \end{array} $$

But the issue in Eq. (23) is that *m* must be very large in order to achieve the accurate estimate of *α*. In this work, we choose the second way, which is the maximum likelihood estimation. To do so, we need to assume that the processes {*I*_*i*_(*t*);0≤*t*≤*T*} (*i*=1,2,⋯,*m*) are mutually independent. Then the log-likelihood function of {*I*_*i*_(*t*);0≤*t*≤*T*} (*i*=1,2,⋯,*m*) is given by

25 $$\begin{array}{@{}rcl@{}} &&\sum\limits_{i=1}^{m}\log f(\alpha|I_{i})\varpropto\\ &&-\sum\limits_{i=1}^{m}\sum\limits_{k=0}^{n-1}\frac{(\triangle I_{i}(k)-(\alpha+\widehat{\delta}_{i}I_{i}(t_{k}))\triangle t(k))^{2}}{2\triangle t(k)\widehat{{\sigma_{i}^{2}}}}. \end{array} $$

The maximum likelihood estimate is

26 $$\begin{array}{@{}rcl@{}} \widetilde{\alpha}=\sum\limits_{i=1}^{m}\omega_{i}\widehat{\alpha}_{i}, \end{array} $$

where

27 $$\begin{array}{@{}rcl@{}} \omega_{j}&=&\frac{\widehat{{\sigma_{j}^{2}}}^{-1}}{\sum\limits_{i=1}^{m}\sum\limits_{k=0}^{n-1}\widehat{{\sigma_{i}^{2}}}^{-1}},\,\,j=1,2,\cdots,m. \end{array} $$

$\widehat {\alpha }_{i}$ is defined in Eq. (24).

## Results and discussion

In this section, we illustrate the performance of our proposed model using both simulated and real data.

### Simulation study

In the simulation study, we examine the performance of our proposed model with respect to the accuracy of parameter estimation and the forward prediction of the case incidence. First, we generate data using various parameters by the following steps:

- Set *m*=4 (the number of sub-populations) and *T*=100 (the number of time slots). These two numbers are randomly selected.
- Randomly draw *α* from [0.05,0.09], *δ*_*i*_ from [0.02,0.08], and *σ*_*i*_ from [0.02,0.08].
- Initialize *I*_*i*_(0),1≤*i*≤*m*.
- Simulate *I*_*i*_(*k*+1)=*I*_*i*_(*k*)+△*I*_*i*_(*k*),*k*=0,1,⋯,*T*−1 using Eq. (7) and $\triangle I_{i}(k)|I_{i}(t_{k})\sim N\left ((\alpha +\delta _{i}I_{i}(t_{k}))\triangle t(k),{\sigma _{i}^{2}}\triangle t(k)\right)$.

Three parameters, *α*,*δ*_*i*_, and *σ*_*i*_, in Eq. (2) will be estimated from the simulated data. We conduct 100 replicates by repeating Step 2–4 and compare the estimated ones, $\hat {\alpha }, \hat {\delta _{i}},$ and $\hat {\sigma _{i}}$, with the ground truth values in terms of the mean absolute percentage error (MAPE) defined as:

28 $$\begin{array}{@{}rcl@{}} E_{\alpha} = \frac{1}{100}\sum\limits_{j=1}^{100}\left|\frac{\hat{\alpha}_{j} - \alpha_{j}}{\alpha_{j}}\right|, \end{array} $$

29 $$\begin{array}{@{}rcl@{}} E_{\delta} = \frac{1}{100*m}\sum\limits_{i=0}^{m-1}\sum\limits_{j=1}^{100}\left|\frac{\hat{\delta}_{ij} - \delta_{ij}}{\delta_{ij}}\right|, \end{array} $$

30 $$\begin{array}{@{}rcl@{}} E_{\sigma} = \frac{1}{100*m}\sum\limits_{i=0}^{m-1}\sum\limits_{j=1}^{100}\left|\frac{\hat{\sigma}_{ij} - \sigma_{ij}}{\sigma_{ij}}\right|. \end{array} $$

**Fig. 1:**
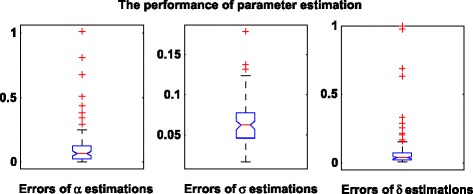
The performance of parameter estimation. The mean absolute percentage errors for *α*, *δ*, and *σ* are 10.0, 6.0, and 10.0 *%*, respectively. *α* measures the auto-recovery rate of one particular infectious disease. *δ* measures the disease transmissibility within the population. *σ* measures the disease transmissibility between populations

**Fig. 2:**
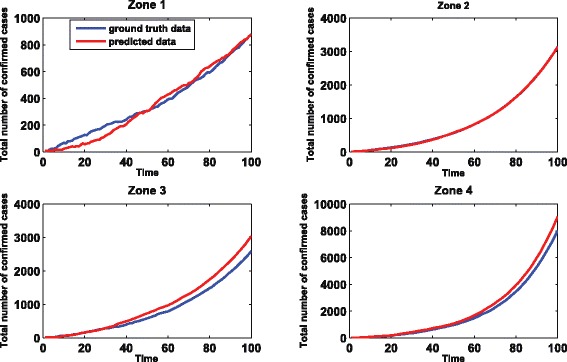
The comparison of original data and estimated data for four regions. The x-axis represents the time in days. The y-axis represents the total number of confirmed cases. The The original data is generated using the ground truth values of parameters while the estimated data is generated with estimated values of parameters. The correlation between them is 0.96

The mean absolute percentage errors (MAPEs) for *E*_*α*_, *E*_*δ*_, and *E*_*σ*_ are 10.0 *%*, 6.0 *%*, and 10.0 *%*, respectively. We plot the distribution of the estimated errors for 100 replicates for $\hat {\alpha }, \hat {\delta _{i}},$ and $\hat {\sigma _{i}}$ in Fig. 1. From Fig. 1, we can see that both the estimates of $\hat {\alpha }$ and $\hat {\sigma _{i}}$ have small variations. The variation of the estimate of $\hat {\delta _{i}}$ is slightly larger but is still acceptable and is due to the uncertainty embedded in the stochastic process. We also use the estimated values of the parameters to generate the data and compare it with the simulated data using the ground truth values of the parameters. The correlation between them is 0.96. We randomly select one replicate and show the comparison results in Fig. 2. Basically, we can use the estimated parameters to accurately recover the ground truth data.

Next, we conduct an experiment to test the prediction accuracy of our model. Let us consider a sequence of data points *I*_*ij*_(*t*) over a time interval [0,*T*] for the *i*_*th*_ subpopulation in the *j*_*th*_ replicate. We choose a time point *s* and use the data points *I*_*ij*_[0],*I*_*ij*_[1],⋯,*I*_*ij*_[*s*] as the training data and predict the data points *I*_*ij*_(*t*) for *s*<*t*≤*T*. *s*=80 is chosen in this experiment. The MAPE of the prediction is defined as

31 $$\begin{array}{@{}rcl@{}} E_{pre} = \frac{1}{100*20*m}\sum\limits_{j=1}^{100}\sum\limits_{i=0}^{m-1}\sum\limits_{s=81}^{100}\left|\frac{\hat{I}_{ij}[t] - I_{ij}[t]}{I_{ij}[t]}\right|. \end{array} $$

**Fig. 3:**
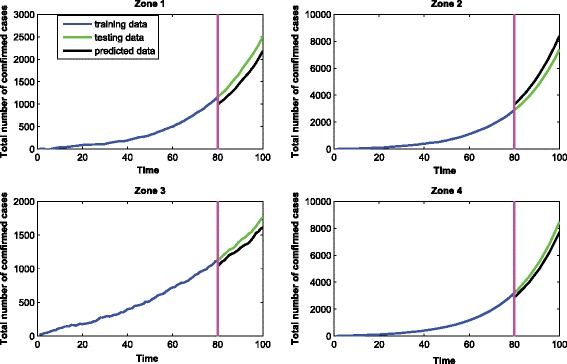
The prediction performance of our method using simulation data for four regions.The x-axis represents the time in days. The y-axis represents the total number of confirmed cases. The data for the first 80 days are used for training. The data for the last 20 days are used for testing. The mean absolute percentage error in testing is 17.3 *%*

The MAPE of the prediction is 17.3 *%*, which indicates that our model can achieve around 82.7 *%* accuracy in terms of the prediction. Again, we randomly select one replicate and show the prediction results in Fig. 3.

### Real application

**Fig. 4:**
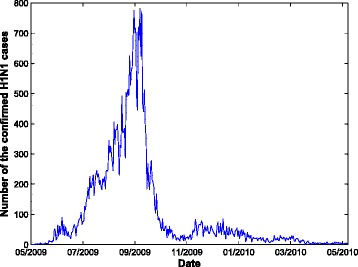
Daily H1N1 epidemic curve in Hong Kong from May 1, 2009 to May 23, 2010. The epidemic curve of confirmed H1N1 cases reaches its peak at the end of September, 2010

In the case study, we apply our model to the surveillance data from the 2009 H1N1 pandemic in Hong Kong. We acquired the time series data of the daily number of confirmed H1N1 cases with symptom onset from May 1, 2009 to May 23, 2010. The database includes 36 547 confirmed cases with demographic information on location, age, and sex along with the laboratory confirmation dates. The epidemic curve of confirmed H1N1 cases (see Fig. 4) reaches its peak at the end of September 2009, after which the intervention procedure comes into effect and the curve goes down. We use the data up to September 30, 2009, which comprises 27 898 cases (more than 2/3 of all cases). Hong Kong is geographically divided by 18 political areas (districts). Each district is considered as one sub-population in our proposed model. The time interval △*t*(*k*)(*k*=0,1,⋯,*n*−1) of H1N1 is set as 1 day. The total number of days is 100.

**Fig. 5:**
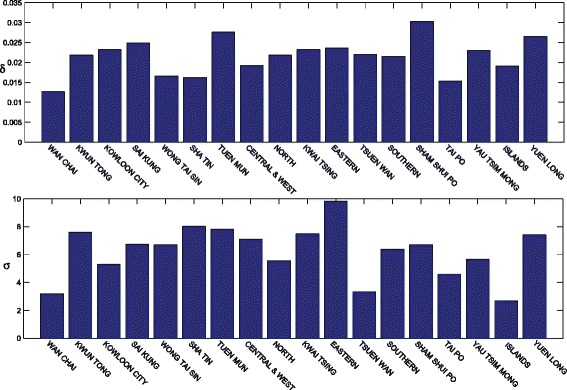
The estimation of two factors in disease spread for 18 districts for 2009 H1N1 pandemic in Hong Kong. *δ* measures the transmissibility of disease spread within each district. *σ* measures the transmissibility of disease spread from the neighbors of each region. This figure shows that the imported infections for each district account for a critical factor in the disease spread while the internal effects only play a very small role in the progression of disease spread

Figure 5 gives the effect of the different components for the 18 political areas in Hong Kong. From Fig. 5, we can find that the effect of *δ*, which measures the internal disease spread within each district, varies less than the effect of *σ*, which measures the external disease spread between districts. In general, the speed of internal disease spread is closely connected with the population density and the external disease spread pattern is associated with the pattern of people’s daily travel. It is well known that Hong Kong has the highest population density in the world, and most districts are densely populated. However, it possesses a heavy heterogeneous traffic pattern, and there is intensive traffic between districts every day. Therefore, the imported infections for each district account for a critical factor in the disease spread, whereas the internal effects only play a very small role in the progression of disease spread.

**Fig. 6:**
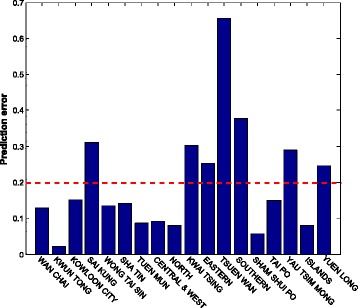
The prediction errors for 18 districts using the real H1N1 data. The mean absolute percentage error is 20.0 *%*

**Fig. 7:**
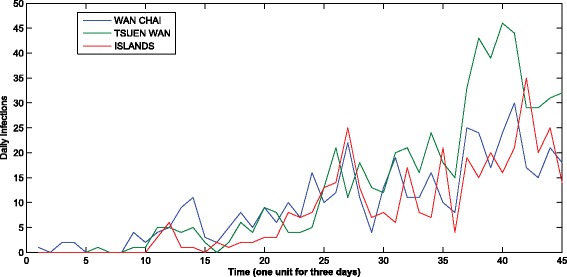
The epidemic curve of three districts with the lowest incidence rates. The x-axis represents the time staring from May 1, 2009 to May 23, 2010. Between time slot 34 and 42, there is a sudden rise for the “TSUEN WAN” district. Such a change significantly affects the parameter estimation and thereby the prediction accuracy for the district “TSUEN WAN”

We also use the H1N1 data to test the prediction accuracy of our model. The MAPEs for all districts are shown in Fig. 6 and Table 1. The average prediction error is 20.0 *%*. We notice that the prediction error for the district “TSUEN WAN” is very high because the number of daily infections in this district changes suddenly during the epidemic period. Figure 7 shows the epidemic curves of the three regions with the lowest incidence rate. We can observe that between time slot 34 and 42, there is a sudden rise for the “TSUEN WAN” district. Such a change significantly affects the parameter estimation and thereby the prediction accuracy for the district “TSUEN WAN”. Although the incidence rates of the other two districts also low, their epidemic curves are relatively smooth in comparison with that of “TSUEN WAN”, indicating that the prediction accuracies of these two districts are higher than that of the “TSUEN WAN” district.

**Table 1:** Prediction error for real data

District	WAN CHAI	KWUN TONG	KOWLOON CITY	SAI KUNG	WONG TAI SIN	SHA TIN
X Error	0.12	0.02	0.15	0.31	0.13	0.14
District	TUEN MUN	CENTRAL &WEST	NORTH	KWAI TSING	EASTER	TSUEN WAN
Error	0.09	0.09	0.08	0.30	0.25	0.65
District	SOUTHERN	SHAM SHUI PO	TAI PO	YAU TSIM MONG	ISLANDS	YUEN LONG
Error	0.38	0.06	0.15	0.29	0.08	0.25

The average error of 18 districts is 0.20

## Conclusions

Epidemic modelling offers a practical means for policy makers to evaluate the potential effects of intervention strategies. To do so, the accuracy of epidemic modelling with respect to the real-world disease transmission dynamics is essential and remains a challenging task due to the inaccessibility of many factors that affect the spread patterns of infectious diseases. In particular, heterogeneity should be taken into consideration when modelling the disease spread in non-random mixing populations. Many methods have been proposed to deal with heterogeneity in the study of epidemic dynamics, mostly using network-based epidemic models in which nodes correspond to spatial locations with reported incidences over time, and the directional links indicate the probability of disease transmission from one node to another over time. However, it is very challenging to determine the network topology. Many studies have used a geographical topology whereas others have used a mobility network inferred from the public transportation network or other sources. How to verify the inferred network topology is another challenging issue because the true epidemic network topology is unknown, and it may vary for different types of infectious diseases for the same population. Furthermore, the neighborhood effect estimation is non-trivial; it involves many parameters (a polynomial of the number of nodes) and requires a large amount of data to avoid overfitting. Such data may not always be available for the inference of network topology. Therefore, in this work, we propose an alternate approach to investigate the spatial heterogeneity from temporal-spatial surveillance data without the inference of network topology.

Our proposed model possesses several merits over the previous works. First, it quantifies the role of the heterogeneity in the analysis of the spread dynamics of infectious diseases in heterogeneous populations. Second, parameter estimation can be computed very quickly. Therefore, the prediction and the corresponding intervention policies can be implemented without delay in an outbreak of infectious disease. We apply our model on both the simulated data and the real data from the 2009 H1N1 epidemic in Hong Kong and achieve acceptable prediction accuracy. Based on the study of disease diffusion, the model proposed in this work can be extended to study other propagation patterns such as the Internet and World Wide Web, through which individuals form multiple communities in which information can propagate in a manner similar to that of infectious disease. We believe that our work makes theoretical and empirical contributions in many areas.

There are some limitations in our proposed stochastic model. First, it does not consider the epidemic network topology. However, how to infer such networks is another challenging task. To the best of our knowledge, the best way to do so is to use the contact data among some infected patients to verify the results, but such data are not always available and can be difficult to collect due to many issues (e.g., privacy). This issue may be addressed by using other types of data, such as daily commute data extracted from social networks. Second, our proposed model achieves a prediction accuracy of only around 80 *%*. We need to further improve it to allow its full use in real applications. Third, the proposed model is only suitable for the situation in which the susceptible population (or sub-population) maintains a relatively constant size and structure in a region. However, if the number of infected people in an epidemic is large or asymptomatic infection plays a central role (e.g., the malaria epidemic in Africa), the population factor should be taken into consideration in the model. Moreover, for a highly spatially heterogeneous outbreak (e.g., the Ebola epidemic) in which cases may seem to disappear due to reduced transmission in one area while growth may continue or rise in new locales, our proposed model may have problems in capturing these opposite dynamics in different regions. Fourth, because the proposed model is based on the classic SIR model, it only works in the situation in which the number of infected people grows exponentially. We will investigate resolutions to these limitations in our future work.

## Additional file

Additional file 1 Multilingual abstracts in the five official working languages of the United Nations. (PDF 598 kb)
