# Erratum to: Stochastic modelling of infectious diseases for heterogeneous populations

**DOI:** 10.1186/s40249-017-0269-3

**Published:** 2017-02-28

**Authors:** Rui-Xing Ming, Jiming Liu, William K. W. Cheung, Xiang Wan

**Affiliations:** 1 School of Statistics & Mathematics, Zhejiang Gongshang University, Hangzhou, China; 2 Department of Computer Science, Hong Kong Baptist University, Kowloon Tong, Hong Kong; 3 Department of Computer Science & Institute of Theoretical and Computational Study, Hong Kong Baptist University, Kowloon Tong, Hong Kong

## Erratum

After the publication of this work [1], we noticed an error the author’s name Ji-Ming Liu should be spelt as Jiming Liu.

The original article was corrected.

The publisher apologises for this error.
