# Supplementary material for: Stochastic modelling of infectious diseases for heterogeneous populations
Source: Infect Dis Poverty. 2016 Dec 22;5:107. doi: 10.1186/s40249-016-0199-5 (PMC5178099; doi:10.1186/s40249-016-0199-5)
Supplement: Additional file 1 — Multilingual abstracts in the five official working languages of the United Nations. (PDF 598 kb) [file 40249_2016_199_MOESM1_ESM.pdf]

Translation of the abstract into the five official working languages of the United Nations

نمذجة عشوائية للأمراض المعدية للسكان غير المتجانسين

Rui-Xing Ming, Ji-Ming Liu, William K.W. Cheung, Xiang Wan

ملخص

يمكن أن تؤثر الأمراض المعدية مثل فيروس سارس ومرض انفلونزا H1N1 بشكل كبير على حياة الأشخاص وتسبب أضرار اقتصادية واجتماعية حادة. وقد أكدت حالات التفشي في الآونة الأخيرة على الضرورة الملحة لإجراء بحوثات فعالة على ديناميات انتشار الأمراض المعدية. ومع ذلك، من الصعب التنبؤ أين ومتى ستظهر حالات التفشي وكيفية انتشار الأمراض المعدية بسبب أن العديد من العوامل تؤثر على انتقالها وقد تكون بعض تلك العوامل غير معروفة. وتعد إحدى الوسائل الممكنة للكشف فورًا على حالات التفشي وتتبع تقدمها هي تنفيذ نظم مراقبة في مرافق الصحة الإقليمية أو الدولية والمراكز الطبية. ويمكن أن توفر بيانات المراقبة المتراكمة بما فيها المعلومات الزمانية والمكانية والسريرية والديموغرافية معلومات قيمة يمكن استغلالها لفهم بشكل أفضل ونمذجة ديناميات انتشار الأمراض المعدية. في هذا العمل، نقترح إجراء نموذج عشوائي لدراسة ديناميات انتشار الأمراض المعدية في المجتمعات غير المتجانسة من خلال بيانات المراقبة الزمانية-المكانية. قمنا باختبار النموذج المقترح على بيانات المحاكاه وطبقناها على بيانات المراقبة على وباء H1N1 الذي تفشى في هونج كونج 2009. في تجربة المحاكاة، حقق نموذجنا دقة عالية في تقييم البارامتر (أقل من 10.0% يعني خطأ مطلق في النسبة المئوية). وفيما يتعلق بالتوقع المستقبلي لحالات الإصابة، فإن الخطأ المطلق في النسبة المئوية بلغ نسبته 17.3% لتجربة المحاكاة ونسبة 20.0% للتجربة على بيانات المراقبة الفعلية. نحن نؤمن بأن نموذجنا يمكن أن يوفر معلومات قيمة لسلطات الصحة العامة للتنبؤ بتأثير انتشار المرض وتحليل العوامل الكامنة وتوجيه جهود المكافحة الجديدة.

Translated from English version into Arabic by Yosra Montasser, through

TRANSLATORS WITHOUT BORDERS

传染病在异构人群中传播的随机模型

明瑞星、刘际明、张国威和万翔

摘要

近年來在全球爆发的传染病疫情如 SARS 和 H1N1 严重影响了人们的日常生活，而且造成重大的经济损失，因此更加迫切的需要研究人员进一步深层次地研究传染病传播机制及有效的预防策略。然而，由于有许多因素包括一些未知因素牵扯在其中，预测何时何地可能出现传染病的爆发以及传染病会以什么方式在人群中传播是非常困难的。一个可行的办法是在区域范围及国家层面的健康和医疗中心建立实时监控系统，以便及时发现流行病的爆发及跟踪疾病的传播。由此，研究人员可以使用长期累计监视数据，包括随时间变化及区域变化的监测数据、临床诊断数据和人口统计信息，来建立新的传染病动态传播模型，从而可以更好地评估和预测传染病蔓延。本文的工作是开发一个随机动态模型用来研究传染病在异构人群中的传播模式。我们使用仿真数据以及 2009 年香港 H1N1 流感大流行的监测数据来测试我们设计的模型。对于仿真数据，我们的模性参数估计精度低于 10%的平均绝对百分比误差(MAPE)。对于模型的预测精度，仿真数据的绝对百分比误差是 17.3%，真实数据的结果是 20%。基于

这些可接受的预测精度，我们相信该模型完全可以被应用于实践中，帮助传染病控制部门分析传染病传播的关键因素、预测疾病扩散的程度并且及时的发布有效的控制措施。

Translated from English version into Chinese by Rui-Xing Ming

Modélisation stochastique de maladies infectieuses pour des populations hétérogènes

Rui-Xing Ming, Ji-Ming Liu, William K.W. Cheung, Xiang Wan

Résumé

Les maladies infectieuses comme le SRAS et le H1N1 peuvent influencer la vie des personnes de manière significative et générer des dommages sociaux et économiques sévères. Les récentes épidémies ont souligné l'urgence d'une recherche effective sur les dynamiques de la propagation de maladies infectieuses. Il est néanmoins difficile de prédire le moment et l'endroit où des épidémies peuvent émerger et la manière dont des maladies infectieuses se propagent en raison de la présence de nombreux facteurs affectant leur transmission, dont certains peuvent être inconnus. L'un des moyens exploitables de détection rapide d'une épidémie et de suivi d'une épidémie consiste à mettre en œuvre des systèmes de surveillance au sein de centres de santé et de centres médicaux régionaux ou nationaux. Les données de surveillance accumulées, y compris les informations chronologiques, spatiales, cliniques et démographiques peuvent fournir des informations précieuses susceptibles d'être exploitées afin d'améliorer la compréhension et de modéliser les dynamiques de la propagation de maladies infectieuses. Dans cet article, nous proposons l'élaboration d'un modèle stochastique destiné à étudier les dynamiques de la propagation de maladies infectieuses au sein de populations hétérogènes à partir de données de surveillance spatio-temporelles. Nous testons le modèle proposé sur des données de simulation et l'appliquons aux données de surveillance provenant de l'épidémie du virus H1N1 de 2009 à Hong-Kong. Dans le cadre de notre expérience de simulation, notre modèle permet d'atteindre une grande précision en termes d'estimation des paramètres (pourcentage d'erreur absolu moyen inférieur à 10,0 %). En termes de prédiction de l'incidence des cas, les pourcentages d'erreur absolus moyens sont de 17,3 % pour l'expérience de simulation et de 20,0 % pour l'expérience sur les données de surveillance réelle. Nous estimons que notre modèle peut fournir des informations exploitables aux autorités de santé publique pour la prédiction de l'effet d'une épidémie et l'analyse de ses facteurs sous-jacents tout en orientant de nouveaux efforts de contrôle.

Translated from English version into French by eric ragu, through

TRANSLATORS
WITHOUT BORDERS

Вероятностная модель инфекционных заболеваний для неоднородных совокупностей

Жуйсин Мин (Rui-Xing Ming), Цзимин Лиу (Ji-Ming Liu), Уильям К. В. Чэун (William K.W. Cheung), Сян Вань (Xiang Wan)

Реферат

Инфекционные заболевания, такие как ТОРС (тяжелый острый респираторный синдром) и H1N1 (подтип вируса гриппа А), могут в значительной степени воздействовать на жизни людей и причинять серьезный социально-экономический ущерб. Недавние вспышки инфекций подчеркнули настоятельную необходимость эффективного исследования динамики распространения инфекционных заболеваний. Однако, трудно прогнозировать время и место возникновения вспышек инфекционных заболеваний и пути их распространения, поскольку на передачу инфекций воздействуют многочисленные факторы, причем некоторые из них могут быть неизвестны. Одним из возможных средств быстрого выявления вспышек и отслеживания распространения заболеваний является внедрение систем наблюдения в региональных и национальных поликлиниках и медицинских центрах. Накопленные данные наблюдений, включая временные, территориальные, клинические и демографические сведения, могут служить источником ценной информации для лучшего понимания и моделирования динамики распространения инфекционных заболеваний. В данной работе мы предлагаем вероятностную модель изучения динамики распространения инфекционных заболеваний в неоднородных совокупностях на основании территориально-временных данных наблюдений. Представленная модель испытывается на данных для моделирования и применяется к данным наблюдения за пандемией гриппа H1N1 в Гонконге в 2009 году. В моделирующем эксперименте наша модель достигает высокой степени точности в оценивании параметров (средняя абсолютная погрешность – менее 10,0%). Что касается прямого предсказания случаев заболевания, средняя абсолютная погрешность составляет 17,3% для моделирующего эксперимента и 20,0% – для эксперимента с реальными данными наблюдений. Мы считаем, что наша модель может быть полезна органам здравоохранения для прогнозирования последствий распространения заболеваний, анализа лежащих в основе этого факторов и определения новых способов контроля.

Translated from English version into Russian by Tatyana Johnson, through

TRANSLATORS
WITHOUT BORDERS

Modelo estocástico de enfermedades infecciosas para poblaciones heterogéneas

Rui-Xing Ming, Ji-Ming Liu, William K.W. Cheung, Xiang Wan

Resumen

Las enfermedades infecciosas como el SRAS y la H1N1 pueden tener un impacto significativo en

la vida de las personas y producir daños sociales y económicos severos. Los brotes recientes han enfatizado la necesidad urgente de una investigación efectiva sobre la dinámica de la propagación de la enfermedad infecciosa. Sin embargo, es difícil predecir cuándo y dónde surgirán los brotes y cómo se propagan las enfermedades contagiosas debido a que son muchos los factores que afectan su transmisión y algunos de ellos pueden no ser conocidos. Una manera viable de detectar rápidamente un brote y vigilar el progreso de la propagación de la enfermedad es la implementación de sistemas de vigilancia en centros médicos regionales o nacionales. Los datos de vigilancia acumulados, incluyendo información temporal, espacial, clínica y demográfica, pueden proporcionar información valiosa que podemos explotar para entender mejor y modelar la dinámica de la propagación de la enfermedad infecciosa. En este trabajo proponemos un modelo estocástico para estudiar la dinámica de la propagación de la enfermedad infecciosa en poblaciones heterogéneas a partir de datos de vigilancia temporo-espacial. Evaluamos el modelo propuesto en datos de simulación y lo aplicamos a los datos de vigilancia de la pandemia de H1N1 en Hong Kong en el año 2009. En el experimento de simulación, nuestro modelo logra alta precisión en la estimación de parámetros (porcentaje de error medio absoluto de menos del 10,0%) En términos de la predicción hacia adelante de la incidencia de casos, el porcentaje de error medio absoluto es de 17,3% para el experimento de simulación y 20,0% para el experimento sobre los datos de vigilancia real. Creemos que nuestro modelo puede ofrecer un valioso entendimiento para que las autoridades de salud pública puedan predecir los efectos de la propagación de la enfermedad y analizar los factores subyacentes y para dirigir nuevos esfuerzos de control.

Translated from English version into Spanish by Maria Alejandra Aguada, through

TRANSLATORS
WITHOUT BORDERS
